# Biographical Feature: Ruth Ella Moore, Ph.D.

**DOI:** 10.1128/jcm.01863-24

**Published:** 2025-02-27

**Authors:** Marian C. Johnson-Thompson

**Affiliations:** 1 Center for History of Microbiology and ASM Archives (CHOMA) 8315 , Washington, DC, USA; 2 College of Arts and Sciences, University of the District of Columbia 8315 , Washington, DC, USA; Marquette University, Milwaukee, Wisconsin, USA

## TEXT

**Figure:**
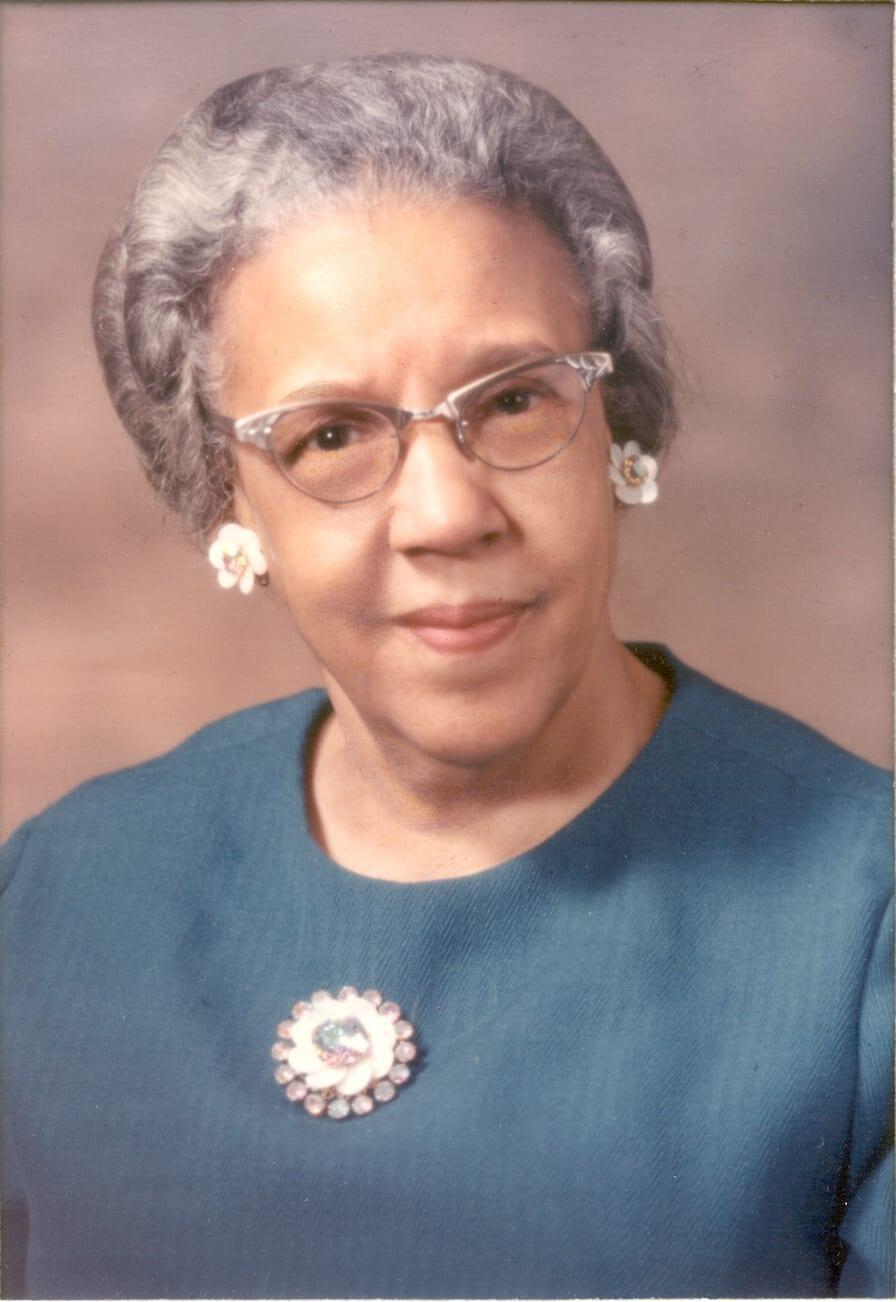


In 1933, Ruth Ella Moore became the first woman and among the first five recipients to earn a Ph.D. in Bacteriology from The Ohio State University (OSU). Additionally, she became the first Black to earn this degree and the first Black female to earn a Ph.D. in the Natural Sciences from any U.S. University. Moore spent her entire career at Howard University Medical School (HUMS) where she was the first woman to chair a department. Conducting a rigorous schedule of teaching medical, nursing, and dental students; preparing laboratories; conducting tuberculosis, dental caries, gut microorganisms, and blood research; and administering departmental matters, she also found time to engage in external professional activities and contribute to her community.

Yet, Moore’s entry is missing from the chronological listing of bacteriology Ph.D. recipients in OSU archives. Similarly, in Margaret Rossiter’s 1982 landmark study, *Women Scientists in America, Volume One—Struggles and Strategies to 1940* (1) Moore is excluded. However, Rossiter’s study suggests that Moore was the fourth American woman to receive a Ph.D. in bacteriology after Ida Bengtson, Sara Braham Matthews, and Margaret Pittman; all three are cited in Rossiter’s text. That the two aforementioned events occurred informs us about the discrimination Moore faced. After earning a Ph.D. from a top-tier university, it also suggests the increased contributions she could have made if equitable intellectual (including mentoring) and physical resources had been available. This biographical sketch is the first detailed compilation of her contributions with a focus on the barriers she faced throughout her training and professional life. Also, it will shed light on her personal and family life, which most probably enabled her to flourish in spite of these barriers.

A mere 40 years after Emancipation, 24 years following the end of Reconstruction, and 61 years before the Civil Rights Act, Moore was born on 19 May 1903, in Columbus, OH, USA. Her parents, Margaret and William E. Moore, were from Virginia and Ohio, respectively. Moore’s father worked in the city tax office, and her mother was an accomplished seamstress having graduated from the Columbus State College of Art and Design. Moore attended the segregated and unequally resourced public schools of Columbus, OH, USA. It was Moore’s mother who emphasized the importance of education and pushed her to excel. Upon graduation from high school, she enrolled in OSU, one of the few non-Black universities that would accept African Americans. Moore’s maternal and paternal grandmothers and her paternal grandfather were born in Virginia. It is probable that they were enslaved in Virginia, and because Ohio was a free state, it was also a destination for enslaved Blacks seeking freedom.

Moore earned the BA and MA degrees in 1926 and 1927, respectively, from OSU. Her MA thesis was *A study of some factors which modify the determination of the phenol coefficient*. Afterward, she accepted employment at Tennessee State College (now Tennessee State University) from 1927 to 1929. After “teaching everything from English to hygiene” (2), with no opportunity to teach bacteriology, she was motivated to enroll in OSU to pursue a Ph.D. in Bacteriology. She earned the degree in 1933; her dissertation was *Part I. Studies on dissociation of Mycobacterium Tuberculosis* and *Part II. A New Method of Concentration of the Tubercle Bacilli as Applied to Sputum and Urine Examinations*. Moore studied under William A. Starin, Ph.D., who joined the department in 1910 as an instructor, was promoted to full professor in 1918, and earned a Ph.D. from the University of Chicago in 1923 (3).

During the post-Depression Era, tuberculosis was the second leading cause of death due to an infectious disease in the U.S., though the rates were declining (4). Apparently, Moore’s thesis was or could have been important in contributing to identifying and eradicating tuberculosis. Moore’s research on *Mycobacterium tuberculosis* was never published. Yet, Starin was active in publishing while at OSU, active in the American Society for Microbiology (ASM) and ASM’s Columbus, OH chapter, and later served as department chair. Records show that most of Starin’s publications were dated immediately prior to Moore’s Ph.D. matriculation. However, while Starin’s publications reflected his research with *Clostridium botulinum*, Moore’s research was on *M. tuberculosis*. In fact, when one looks at the publication record of each faculty member around the time that Moore matriculated, no one in the department worked with *Mycobacterium*. This manner of a graduate student’s thesis lacking relevance to the advisor or any other departmental faculty’s research is unusual.

In *OSU’s History of the Bacteriology Department from 1873 to 1969* (5), the department was organized in 1903. From 1920 to 1935 only five total Ph.D.s had been granted. Moore would have been among those first graduates and the first woman to earn a Bacteriology Ph.D. from OSU if one genderizes the names. (Incidentally, it would not be until 1938 that the next woman, a white woman, would earn the Bacteriology Ph.D. from OSU. Her thesis was published.) Furthermore, the listing of OSU Ph.D. bacteriology recipients from 1920 to 1934 cites only three graduates. According to the current Chair of OSU’s Bacteriology Department, Kurt Frederick, Ph.D., “some data from the early years are fragmented” (personal communication). Though Rossiter excludes Moore’s name, her data show that by 1938, two women had earned a Bacteriology Ph.D. from OSU. Nevertheless, from Rossiter’s citations (1), one can assume that Moore was the first American woman to receive a Ph.D. in Bacteriology from OSU and the fourth American woman to receive a Bacteriology Ph.D. from any U.S. university. Rossiter’s follow-up text (6) shows that another woman would not earn a Ph.D. in bacteriology until Esther Lederberg did so in 1950 from the University of Wisconsin. Of note, Rossiter assembles a table of the first Black women Ph.D. scientists from Vivian Sammons’ book, *Blacks in Science and Medicine* (7). Moore’s name is captured, but that is the extent to which Rossiter includes her.

From the beginning of Moore’s matriculation, racism prevented her from achieving all that she could have accomplished. Though OSU allowed her enrollment, one can imagine that segregated housing, dining, library, cafeteria, and restroom facilities were barriers, which hindered her full academic, social, and comfortable participation. OSU dormitories did not integrate until 1946 (8). A 1926 OSU student handbook reveals that Moore lived near but not on campus. Thus, Moore would commute daily. According to Sandra Jamison, Moore’s cousin, this was not the family home but probably a segregated off-campus rooming house that provided closer access to OSU.

Indeed, during Moore’s educational and living experiences, segregation was the order of the day. All recreational, health care, religious organizations, etc., were segregated. Typically, and on a large campus as OSU, facilities for Black students were in distant locations and uncomfortable to reach during close scheduling and harsh weather conditions. In 1932, Doris Weaver, a Black woman, took OSU to the Ohio Supreme Court for not allowing her to live in a campus dorm, but the court ruled in favor of OSU, upholding its “separate but equal status” (8). Arthur Webb, Ph.D., described similar barriers (personal communication). Webb earned a Ph.D. in Bacteriology from the University of Illinois in 1944 and is the ninth Black person to receive a Microbiology Ph.D. (9).

During his matriculation, he was not allowed to enter some classes and was forced to sit outside. Additionally, he was never assigned a lab partner. Such practices, indeed, were severe impediments to Webb’s achievement and self-worth and led to extreme bitterness.

Despite the presence of such overt discrimination, Moore demonstrated tremendous motivation and drive and was successful in earning three degrees. This was during the Great Depression when college enrollment declined. Perhaps there was an entity, in addition to her parents, that provided support. Indeed, Moore was a member of Delta Sigma Theta Sorority, Inc., founded in 1913 at Howard University. In 1919, a fifth chapter was chartered at OSU, and membership required the highest academic standards and a commitment to sisterhood and service. It is likely that the sorority provided Moore with a feeling of belonging and the support to excel.

Following Moore’s graduation, she joined the newly reorganized Department of Bacteriology, Preventive Medicine and Public Health at HUMS, in 1933, as an instructor. One could assume that Moore, with a Ph.D. and prior university teaching experience, would have been hired as an Assistant Professor with a wide range of academic employment opportunities. Despite such accomplishments, HUMS and Meharry Medical College might have been the only institutions where she could have applied her talents. Also of note is that of the first nine African Americans who earned Bacteriology Ph.D.s from top-tier white Ph.D. granting institutions, all were able to find employment only at Historically Black Colleges or Universities (HBCU). Unfortunately, these HBCUs lacked the necessary resources for them to continue the type of research for which they had been trained, and during this era, no HBCU offered a Ph.D. in Bacteriology. Other opportunities would have included teaching at an HBCU or secondary school lacking a bacteriology curriculum. Indeed, this was the plight of Edward Bouchet, Ph.D. (1852–1918), the first African American to earn a Ph.D. from an American University (10). In 1876, Bouchet earned a Ph.D. in Physics from Yale University but was unable to secure a university teaching or research position due to discrimination. He took a position teaching precollege courses at the Institute for Colored Youth in Philadelphia, PA, USA (10), where he taught chemistry and physics. In 1902, after 26 years, he was forced to resign when a new all-white board preferred industrial education and eliminated the precollege program.

When Moore joined HUMS, Hildrus Poindexter, M.D., M.A., M.P.H., Ph.D., was Acting Chairperson. Moore was assigned a heavy load of teaching medical, dental, nursing, and dental hygiene students. With no assistance, Moore was responsible for bacteriology lectures and laboratories to include media preparation and laboratory set-ups. Interestingly, in his autobiography, Poindexter does not mention that he recruited and hired Moore. Here, it is important to note that Poindexter received the M.D. degree from Harvard in 1929 and the M.A. and the Bacteriology Ph.D. in 1930 and 1932, respectively, from Columbia University. The latter degree was earned over a 2-year period while Poindexter was in internship and residency training and working part-time at Howard. According to his autobiography (11), the Ph.D. was in “pure science from the Graduate Faculty at Columbia University (11).” The thesis was described as containing only six pages, chiefly tables, and the voluminous amount of data presented required endless amounts of laboratory investigations and analyses. Indeed, there was a School of Pure Science during Poindexter’s enrollment. However, in 1979, the schools of Political Science, Philosophy, and Pure Science merged to form the new Graduate School of Arts and Sciences. As a result, and though Poindexter’s research spanned the specific areas of parasitology, immunology, and epidemiology, the status of being the first black to earn a microbiology Ph.D. degree is appropriately accorded to Moore because she was the first to earn and complete a traditional Ph.D. degree from a bacteriology department (9).

In 1939, Moore was promoted to Assistant Professor. From 1947 to 1948, Moore assumed the position of acting chair and served as chair from 1949 to 1958. Thus, Moore became the first female faculty member to chair a department within HUMS. During this time, Moore’s leadership resulted in many successes that highlight her contributions as a researcher, an instructor, and an administrator in Microbiology. Among them were (i) improved Bacteriology performance on students’ medical license exams, (ii) the department separated into a stand-alone Bacteriology Department, (iii) petitioning the Dean to change the department name to Microbiology (in keeping with current trends and preceding ASM making the change from Society of American Bacteriologists [SAB] to ASM), (iv) initiating a modest research program addressing tuberculosis, tooth decay (12), gut microorganisms (13), and blood types (14, 15), with Moore publishing on these topics, and (v) chairing multiple department committees from 1943 to 1969 and active involvement in local and national ASM activities and participation in other professional organizations. Because of this, she received the tagline “Ma Moore” (in her absence) due to her sincere and welcomed care and her wise mentoring. Despite these achievements, Moore jested to Jamison about her 1934 experience as a faculty member attending her first HU commencement. A seat had not been prepared for her, and she was forced to sit among students. Subsequently, she continued that practice until her 1971 retirement.

An excerpt from a 1967 publication (16), that Moore co-authored with then-chair of the microbiology department, Charles Buggs, Ph.D., describes Moore as a laboratory technician and says nothing about her lecturing duties nor her research studies. She was promoted to Associate Professor in 1960 and indeed developed curriculum materials and lectured medical, dental, and nursing students. In fact, despite her heavy teaching load and laboratory preparations, she had managed to author or co-author four publications. Two were with Madison Briscoe, Ph.D., whose research was described in the article, but Moore’s name was excluded from this collaborative effort. The exclusion is as curious as the fact that Moore is a co-author. One has to wonder why a professional of Moore’s caliber is only cited as preparing laboratories. One also wonders how these omissions that strongly suggest sexual discrimination impacted Moore. Again, she was unseen and unappreciated for her significant contributions.

In the same Journal, Paul Cornely, M.D., a former chair of the combined Department of Bacteriology, Preventive Medicine and Public Health and Chair of the new Department of Preventive Medicine and Public Health, described the evolution and transition of the new department (17, 18). He cited all former chairs through 1967 but never cited Moore. Though the dates span Dr. Moore’s tenure as chair and Cornely’s tenure under her (Cornely had been at the institution since 1934), he never mentions her name in the article. Even in the department where, by this time, she had spent almost 35 years of her professional life, she continued to be unseen and unrecognized by her peers.

With many work responsibilities, Moore attended the 1936 SAB (now ASM) annual meeting in Indianapolis, IN, USA, and was the first African American to do so. Though the earliest available roster of the ASM’s membership ledger shows that William A. Hinton, M.D., paid membership dues as early as 1921, Hinton never attended an ASM meeting (9, 19). Additional ASM records show that Moore and Poindexter were registered for the 1937 meeting held in Baltimore, MD, USA. Moore and Poindexter commuted daily from Washington, DC to Baltimore. Baltimore hotels did not begin to integrate until 1958, while Washington, DC hotels began to integrate after the 1953 Supreme Court decision that ended legal segregation in public accommodations. Poindexter joined the Society in 1944. Although he and Moore were regular attendees of annual meetings from 1937 until retirement, Moore did not become a member until 1947. When meetings were held in the South, Black attendees were unable to fully participate due to prohibition on hotel accommodations, eating in restaurants, and riding in elevators at meeting sites. Most African American members refused to attend. Moore did not attend the 1956 meeting that was held in Houston, and following membership complaints (9), the ASM refrained from meetings in the South until 1969 when hotels in Miami, FL, USA, were integrated. Moore routinely attended the DC Branch of ASM meetings as per the prompt reports she made, which are now located at ASM’s Center for the History of Microbiology and ASM Archives (CHOMA).

In 1986, a contingency of African American members invited Moore to their annual “get-together,” which the ASM refused to financially support, though some ASM leadership attended. The “get-together” was held during the annual ASM General Meeting. It was at this time that African American microbiologists and some ASM staff presented Moore with a Lifetime Achievement Award. At that time, she had only been known to Howard University and to the many students—mostly medical and dental—whom she had trained during her 40 years at HUMS, as well as two entities at OSU. In 1970, OSU awarded Moore the Alumni Centennial Award, and in 1984, the Distinguished Alumni Award. Shortly after the 1986 gathering, ASM began to actively support minority inclusion efforts and to recognize Moore (9). Thus, recognition of Moore slowly increased within the ASM, and the ASM became the primary conduit for promoting Moore’s recognition into the broader microbiology, science, women, and lay audience communities. Moore was also a member of the American Association for the Advancement of Science, the American Association of University Professors, the American Public Health Association, and The New York Academy of Sciences.

Moore never married and had no children. Jamison was her closest relative, though she was 40 years Moore’s junior. Jamison remembers visiting Moore at her home in Washington, DC and attending operatic performances with Moore during her stay. Moore loved opera and ballet and would routinely purchase season tickets to performances. During one particular family visit in the 1950s, Jamison recalls visiting Moore’s laboratory and was horrified when she saw a huge cockroach. Undoubtedly, this was a loose *Blaberus* caniifer *Burmeister* of Moore’s collaboration with Briscoe (13). Jamison had a right to be horrified because this cockroach species is twice the length of the familiar American cockroach.

Jamison also remembers that Moore enjoyed reading, had “many” books, and enjoyed the many international students with whom she liked to entertain with meals at her home. She also had a fondness for inviting friends to dinners that she enjoyed preparing. Influenced by her seamstress mother, Moore was an excellent seamstress. She made her own patterns for her very fashionable outfits, but there is no record of her ever having formal instruction in design and dressmaking. However, one can relate the accuracy used in design and sewing to the precision required in developing protocols and the accuracy required to perform research. Her youthful photos, especially those from her years at OSU and her graduation celebration, reveal a very beautiful and elegantly attired woman. Many of her outfits were preserved and donated to OSU’s School of Textiles and Design, and in 2009, several were featured in *The Sewer’s Art: Quality, Fashion and Economy*, as part of a Sewers Art Exhibit. In 1958, Moore joined Augustana Lutheran Church in Washington, DC, where she was active in all facets of parish life. As a member of the National Council of Churches, she chaired the Women’s ministry.

Some HUMS data cite Moore as retiring after nearly 40 years of service. However, a retirement program recently located at HU’s Moorland-Spingarn Research Center is dated 31 May 1971. This indicates that she retired after 38 years. It is likely that Moore returned and provided intermittent contractual services until 1973. Her last return was in 1987 when she accepted the “Messenger Award.” In 1993, Moore was a guest of honor at Augustana Lutheran Church’s 75th anniversary celebration, and she shared memories about her life in the church and at work. In her own words, which were later written in her obituary, she said, “I have lived a life of peace and enjoyment, loving my family, friends, church and all.” Moore passed from heart failure at the Lutheran Home on 19 July 1994. Augustana Lutheran Church conducted her services at the HU Rankin Chapel, and she rests within her family’s plot, section 86, at the Greenlawn Cemetery in Columbus, OH, USA. Moore’s obituaries appeared in the Washington Post and the ASM News.

Moore’s legacy can be found in the thousands of students she trained in microbiology working in biomedical fields around the world. During her time, and prior to integration, Howard was the destination for students of color from all over the world who pursued a biomedical career. Moore received scant recognition from her Howard peers and none from the broader microbiology community. However, she was highly revered, often with humor, by her students, as noted in several Bison (Howard University yearbook) entries. In 1940 and 1955 entries, Moore is identified as creating “‘weird’ practicals,” and “likes to give little quizzes at the most unexpected times,” yet referred to as self-reliant and amiable. In 1949, she received additional commendations, and a specific entry indicated that her previously designated “Mother Moore” title had been modified to “Ma Moore.”

More than 25 years following Moore’s death, it can be said that Moore received the ultimate award from OSU. In 2021, Moore was posthumously inducted into OSU’s Office of Diversity and Inclusion Hall of Fame. The Ruth Ella Moore Scholarship was established in 2021 with a triple-match campaign to raise $10,000. OSU’s Senior Director of Development was astounded when donors met the 5-year, $100,000 goal in just weeks (20).

Moore was a very proud and modest woman who did not seek recognition. She pursued her tasks uncomplainingly and with grace, fortitude, and tenacity. When one considers the doubly racist and sexist environment in which she navigated, one has to hail her as an exceptional achiever with utmost resilience. That she was able to achieve and contribute while facing racist and sexist barriers is the reason she receives so many delayed accolades. While these barriers should not have occurred, she serves as a symbol that they did, and that society can do better where similar barriers exist today.

## EDITOR'S NOTE

**Ed. Note**: In this and future years, the *Journal of Clinical Microbiology* seeks to broaden the scope of the Biographical Feature by leveraging resources of the Center for the History of Microbiology and ASM Archives (CHOMA) to include occasional vignettes on more historic, contributing medical microbiologists. In this feature (the publication of which is scheduled to correspond with Black History Month), Dr. Marian C. Johnson-Thompson, CHOMA member and the 2019 recipient of the ASM William A. Hinton Award for Advancement of a Diverse Community of Microbiologists, highlights the career of Dr. Ruth Ella Moore. In this well-researched publication, Dr. Johnson-Thompson not only discusses the impact of both racism and sexism on the career path of a talented microbiologist/scientist but also attempts to accurately chronicle sentinel events in Dr. Moore’s life versus what has been purported in online venues.
